# The functional status and well being of people with myalgic encephalomyelitis/chronic fatigue syndrome and their carers

**DOI:** 10.1186/1471-2458-11-402

**Published:** 2011-05-27

**Authors:** Luis C Nacul, Eliana M Lacerda, Peter Campion, Derek Pheby, Maria de L Drachler, José C Leite, Fiona Poland, Amanda Howe, Shagufta Fayyaz, Mariam Molokhia

**Affiliations:** 1 Department of Nutrition and Public Health Interventions Research, London School of Hygiene and Tropical Medicine (LSHTM), Keppel Street, London, WC1E 7HT, UK; 2 Foundation for Genomics and Population Health (PHGF), Cambridge, CB1 8RN, UK; 3 Hull-York Medical School, University of Hull, Hull, HU6 7RX, UK; 4 National ME Observatory Project Coordinator, and Buckinghamshire New University, Society and Health, Uxbridge Campus, 106, Oxford Road, Middlesex, UB8 1NA, UK; 5 University of East Anglia, Norwich, UK; 6 Department of Primary Care & Public Health Sciences, Division of Health and Social Care Research. Kings College, London, SE1 3QD, UK

## Abstract

**Background:**

Diagnosis of myalgic encephalomyelitis/chronic fatigue syndrome or ME/CFS is largely based on clinical history, and exclusion of identifiable causes of chronic fatigue. Characterization of cases and the impact of interventions have been limited due to clinical heterogeneity and a lack of reliable biomarkers for diagnosis and outcome measures. People with ME/CFS (PWME) often report high levels of disability, which are difficult to measure objectively. The well being of family members and those who care for PWME are also likely to be affected. This study aimed to investigate the functional status and well being of PWME and their lay carers, and to compare them with people with other chronic conditions.

**Methods:**

We used a cross sectional design to study 170 people aged between 18 and 64 years with well characterized ME/CFS, and 44 carers, using SF-36 v2™. Mean physical and mental domains scores (scales and component summaries) were calculated and compared internally and externally with reference standards for the general population and for population groups with 10 chronic diseases.

**Results:**

SF-36 scores in PWME were significantly reduced, especially within the physical domain (mean norm-based Physical Component Summary (PCS) score = 26.8), but also within the mental domain (mean norm-based score for Mental Component Summary (MCS) = 34.1). The lowest and highest scale scores were for "Role-Physical" (mean = 25.4) and "Mental Health" (mean = 36.7) respectively. All scores were in general lower than those for the general population and diseased-specific norms for other diseases. Carers of those with ME/CFS tended to have low scores in relation to population norms, particularly within the mental domain (mean = 45.4).

**Conclusions:**

ME/CFS is disabling and has a greater impact on functional status and well being than other chronic diseases such as cancer. The emotional burden of ME/CFS is felt by lay carers as well as by people with ME/CFS. We suggest the use of generic instruments such as SF-36, in combination of other objective outcome measurements, to describe patients and assess treatments.

## Background

The diagnosis of myalgic encephalomyelitis/chronic fatigue syndrome (ME/CFS) requires the presence for over 6 months of fatigue and other symptoms, and restriction of the ability of those affected to sustain previous levels of social, work and leisure activities [1-3]. Thus, by definition, ME/CFS involves some degree of disability, defined as "any restriction or lack (resulting from an impairment) of ability to perform an activity in the manner or within the range considered normal for a human being" [4].

Although fatigue may be very limiting, a range of other symptoms common in ME/CFS such as pain and cognitive impairment may affect function and lead to limitation of activity and social participation. Disability in ME/CFS (and indeed in other chronic conditions) is multi-dimensional, and therefore generic measures of functional status and well being characterise health status more appropriately than symptom reporting alone.

One of the characteristics of disabling conditions is that their impact may be felt beyond those individuals affected, for example by partners and family members, who may need to spend considerable time caring for their sick relatives, and therefore obliged to sacrifice work and social activities. This can not only subject them to an emotional burden but also adversely affect their own and their families" incomes. There are some studies demonstrating the impact of caring for chronically ill patients on the health of carers [5], but we are not aware of any studies investigating the functional status and well being of those who care for people with ME/CFS. This is an important omission in our understanding of the impact of the disease on the family and limits our comprehension of the needs of those caring for people with ME/CFS (PWME).

This study aimed to measure the functional status and well being of adults with ME/CFS and their lay carers using a standardised reporting questionnaire, and to identify those aspects which are most affected. We sought also to examine the impact of different case definitions on our findings and to compare results for ME/CFS with those achieved by people with other chronic conditions. In addition, we investigated the relationship between patients" scores and those achieved by their carers.

Our main study hypotheses were that functional status and well being of people with ME/CFS are significantly compromised and that quality of life of carers is also affected.

## Methods

This cross-sectional study was undertaken as part of the ME/CFS Observatory Research Programme, and investigated the functional status and well being of people with ME/CFS and their carers. This involved setting up a Disease Register for the study of ME/CFS and a series of epidemiological studies [6]. Other parallel Observatory projects included investigation of the perceptions and illness experience of patients and professionals [7], and the social impact of this disorder [8]. We now plan to enhance the Disease Register by linking it to a disease specific biobank and post-mortem tissue bank [9].

The sampling frame comprised 29 General Practitioner (GP) practices in London, East Anglia and East Yorkshire covering a population of over 143,000. We searched systematically the computerised databases of participating practices to identify patients between 18 and 65 years old who had a GP diagnosis of chronic fatigue syndrome or a related diagnosis. We used GP diagnosis to screen for cases, and reviewed the cases thus identified to determine their compliance with the diagnostic criteria adopted for the study.

Since GPs may refer to cases of ME/CFS by different names, we screened cases that had been diagnosed by GPs with any of the following: chronic fatigue syndrome (CFS), ME, post-viral asthenic syndrome (PVAS), fatigue syndrome (FS), fibromyalgia (FMS), post-infectious encephalitis (PIE) and post-viral fatigue syndrome (PVFS). Patients were considered as potential cases if any of the above diagnoses appeared in their individual electronic medical records, or if they were otherwise referred by their GPs even in the event of they not having been identified by the systematic search. Diagnosis was confirmed if the patient conformed to at least one of the following case definitions, that of the Centers for Disease Control (CDC) in 1994, referred here as CDC-1994 criteria [3], the clinical working case definition established in Canada by an Expert Medical Consensus Panel, known as the Canadian criteria [2], and the Epidemiological Case Definition (ECD) [10]. Assessment for concordance with study case definition was through the completion of a computerized research form listing clinical features, which included a built-in algorithm which determined conformity to case definitions, and hence classified individuals as cases or non-cases, the latter being excluded from the study. Cases were asked to name their lay carer (usually a family member or close friend) if they had one, and to provide them with an invitation to take part in the study.

### Data collection

Standard self-completed questionnaires requesting basic information on personal and demographic parameters were mailed to consenting individuals with ME/CFS, and to their main carers.

A further, longer questionnaire was then sent to confirmed cases, seeking more detailed information, including on clinical and socio-economic variables. Functional status and well being were assessed using the standard form of the SF-36v2™ health survey [11]. The SF-36 has been used in patients with ME/CFS in different settings [12-25].

### Data processing and analysis

The SF-36 health domain scales and component summaries were scored using the Quality Metric scoring software [26]. We used norm-based T scores for the Physical (PCS) and Mental (MCS) Component summary scales. We applied two transformations to the data on the eight health domain scales. These included: i) transformation into a norm-based score (NBS); and ii) standard transformation into a scale ranging from 0 to 100. The scales comprising the physical and mental domains are fully described in the SF-36 manual [11], and are summarised below. The 'Physical Functioning' scale measures performance of physical activities such as running, lifting and carrying groceries, climbing stairs, walking etc. Role-Physical includes measures, for example, of limitation or time reductions in capacity for work or other activities, and the kind of work which can be undertaken. 'Bodily pain' covers the intensity of pain, and the extent to which pain interferes with normal activities. 'General Health' relates to respondents' views and expectations on their health. 'Vitality' relates to energy level and fatigue, and addresses subjective well-being. 'Social Functioning' addresses health related impacts on the quantity and quality of social activity. 'Role-Emotional' assesses the effect of mental health on time spent at work or other activities, and the amount and degree of care devoted to work or the performance of other activities. 'Mental health' covers depression, anxiety, loss of behavioural/emotional control and psychological well being. For all domains, low scores indicate poor results. We used norm-based scores in most analyses. This metric is usually preferred as it enables direct comparisons within and between the health domain scales and the two component summary measures. In all cases, the expected population means are 50 and the standard deviations 10. We also present the results for the health domain on the scale of 0 to 100, to enable comparisons with previous studies that used this scoring system.

All other data were entered onto an Access^®^-based data entry form created specifically for the research, and which enabled cases to be classified according to case definition. Data from the SF-36v2™ and other study forms were merged and exported into Stata-IV 11.1^® ^for Windows software, which was used for the analyses.

For descriptive purposes, we calculated the mean scores for each health domain scale and component summaries, by sex, both for the ME/CFS cases and for their carers. We used the medians to describe grouped data on standard scales (0-100 score), as we observed that the values were not normally distributed. For comparison purposes we used the Student's t-test for continuous data. We also considered minimally important differences (MID), i.e. differences of 3 NBS points, with the exception of 'Role-Emotional' and 'Role-Physical' where differences of 4 and 2 points respectively were required for a difference to be considered important. We investigated the association of scores between cases and their carers by simple linear regression [27]. We then contrasted the results with those for the US general population, and gender-age and disease-specific norms [11].

### Ethical considerations

The study was approved by the Multi-Centre Research Ethics Committee (MREC) in London (06-MRE/02/57), the London School of Hygiene and Tropical Medicine Ethics Committee and the local NHS Research Governance Units in London, East Anglia and East Yorkshire. As this was an interview-based observational study no major ethical issues were anticipated. Informed consent was obtained in all cases. As some participants could tire easily while completing the forms, we encouraged them to pause when they needed to.

### Study size

Participants included 170 ME/CFS cases from 18 to 65 years and 43 named carers of people with ME/CFS. This sample size was adequate to detect SF-36 mean score differences of 0.5 standard deviation between two sub-groups of similar size (e.g. those complying or not with a particular case definition, such as the Canadian criteria) within the sample, with a power of 90% and a significance level of 0.05. This yielded a total sample size of 168. The study power was lower for multiple group comparisons and analyses involving carers.

## Results

Of 278 patients who fulfilled any of the diagnostic criteria, 170 (61.1%) completed the SF-36v2™ instrument and research questionnaire. The median age for all respondents was 51.9 years (Interquartile range (IR) = 40.9 - 57.4). For men, the median age was 53.4 (IR = 47.6 - 60.3) and for women 49.5 (IR = 39.9 - 56.2) (*P *= 0.02). The median age of onset of symptoms, counted from when patients first reported severe fatigue with typical accompanying symptoms, was 41.5 years (IR = 30.4 to 48.3), corresponding to a median duration of fatigue of 10 years (IR = 4.2 to 15) at the time of recruitment. Cases included in the study were similar to non-respondents as regards gender *(P *= 0.2), age-group (*P *= 0.3), ethnicity *(P *= 0.7) and marital status (*P *= 0.3). Response rates were higher in East Yorkshire (70%) than in East Anglia (59%) and London (39%) - *P *< 0.01.

**Figure 1:**
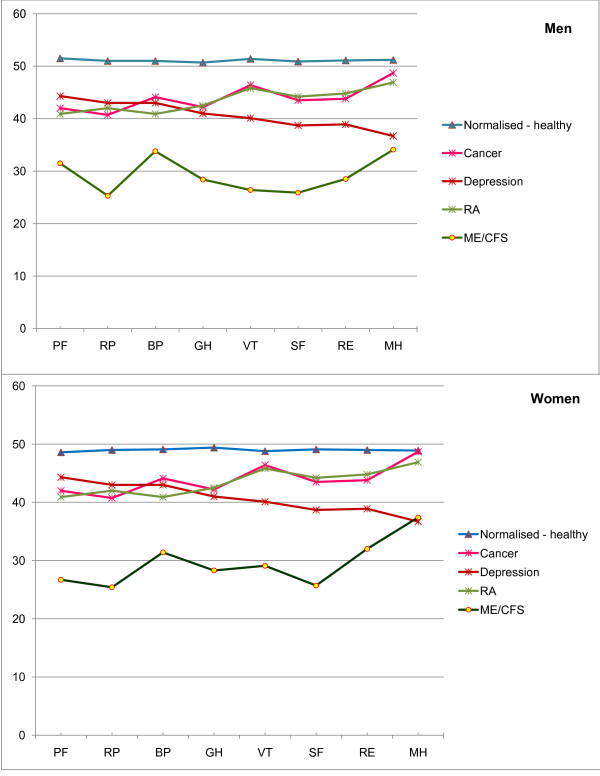
**SF-36v2™ scores in men and women with ME/CFS, other health conditions or healthy**. RA - Rheumatoid arthritis; PF - Physical Functioning, RP - Role-Physical, BP - Bodily pain, GH - General Health, VT - Vitality, SF - Social Functioning, RE - Role-Emotional, MH - Mental health.

Table 1 describes some characteristics of cases and their carers and shows the relationship between them. While 78% of cases were women, the majority of carers (57%) were men. Carers were husbands, wives or partners in 81% of the cases, and a parent or child in 16% (one professional carer was excluded from the analyses). Table 2 summarises the patients' scores, which are presented following norm-based and standard transformations, the latter for comparison with other studies. Norm-based scores within the mental domain tended to be higher than those found in the physical domain (mean MCS = 34.1 and mean PCS = 26.8). Table 3 compares results by gender; in general men scored lower in the mental domain (mean MCS = 30.3 and 35.2 for men and women respectively, *P *= 0.04), and women in the physical domain (PCS = 30.2 and 26.0 for men and women respectively, *P*= 0.02) Mean scores in patients with ME/CFS were consistently lower than population means for 10 other chronic conditions (Table 4). This was true of both sexes (Figure 1). The pattern of higher scores in the physical component scale than those achieved in the mental component scale was consistent with that found in all comparison conditions of predominantly organic origin (e.g. mean PCS = 41.1 and MCS = 47.8 for diabetes), while for depression the opposite was true (mean PCS = 45.4 and MCS = 36.3). Table 5 shows the scores for patients with or without ME/CFS according to the Canadian case definition, and shows consistently lower scores for those meeting the Canadian definition (*P*<0.05 for all scales, except for Role-Emotional). All mean differences were at least minimally important, except in women for the Physical and Mental Component summaries, 'Role-Emotional' and 'Mental Health'.

**Table 1:** Demographic characteristics of the ME/CFS cases and their carers

			Case definition				
					
Demographic variables	ME/CFS cases		CDC-1994	Canadian	ECD	Carers	
	
	n	%	n	n	n	n	%
Gender							
• Male	37	21.8	35	20	7	25	56.8
• Female	133	78.2	131	76	15	19	43.2
	170	100.0	166	96	22	44	100.0
							
Age group							
• 18-24	4	2.4	4	1	2	2	4.5
• 25-34	19	11.2	19	13	2	7	15.9
• 35-44	34	20.0	33	22	4	17	38.6
• 45-54	56	32.9	55	34	7	11	25.0
• 55-64	57	33.5	55	26	7	7	15.9
	170	100.0	166	96	22	44	100.0
							
Ethnicity							
• White British	157	92.9	154	92	19	41	97.6
• Other	12	7.1	11	3	3	1	2.4
	169	100.0	165	95	22	42	100.0
							
Carers' relationship with ME/CFS case							
• Husband/wife/							
partner			35	25	4	35	81.4
• Parent/children			7	6		7	16.3
• Other			1	1		1	2.3
			43	32	4	43	100.0

**Table 2:** SF-36v2™ results in ME/CFS cases

SF-36 scales and summaries	Norm-based scores		Standard scores			
		
	Mean	SD^a^	Mean	SD^a^	Median	IR^b^
Physical Component Summary	26.8	8.4				
• Physical Functioning	27.7	10.6	30.1	24.8	25.0	10.0-45.0
• Role-Physical	25.4	8.2	19.5	20.7	12.5	0.0-31,2
• Bodily Pain	31.9	9.5	28.1	22.4	22.0	12.0-41.0
• General Health	28.3	8.0	24.9	16.7	20.0	10.0-35.0
Mental Component Summary	34.1	11.3				
• Vitality	28.4	7.1	15.5	14.7	12.5	0.0-25.0
• Social Functioning	25.7	9.8	28.9	23.1	25.0	12.5-37.5
• Role-Emotional	31.3	15.1	47.2	32.5	50.0	25.0-75.0
• Mental Health	36.7	12.1	50.9	21.4	55.0	35.0-70.0

^a ^SD = Standard Deviation; ^b ^IR = Interquartile range

**Table 3:** SF-36v2™ results in ME/CFS patients by gender

	Norm-based scores				
		
SF-36 scales and summaries	Males (n = 37)		Females (n = 133)		*P-*value^1^
		
	mean	SD^a^	mean	SD^a^	
Physical Component Summary	30.2	8.8	26.0	8.2	0.023
• Physical Functioning	31.5	13.2	26.7	9.5	0.026
• Role-Physical	25.3	9.2	25.4	7.9	0.840
• Bodily Pain	33.8	10.9	31.4	9.11	0.264
• General Health	28.4	9.7	28.3	7.5	0.993
Mental Component Summary	30.3	11.9	35.2	11.0	0.040
• Vitality	26.4	6.8	29.1	7.1	0.040
• Social Functioning	25.9	11.2	25.7	9.4	0.853
• Role-Emotional	28.5	17.2	32.0	14.5	0.364
• Mental Health	34.0	13.8	37.4	11.5	0.166

^a ^SD = Standard Deviation

^1 ^t-test. For all *P*-values lower than 0.05, except for the vitality score, the difference in means reached the value considered minimally important.

**Table 4:** PCS and MCS mean scores for ME/CFS cases and selected SF-36 disease-specific norms

Disease	Physical Component Summary		Mental Component Summary	
	
	mean	SD^a^	mean	SD^a^
ME/CFS (n = 170)	26.8	8.4	34.1	11.3
Back pain/sciatica (n = 2648)	45.7	10.7	47.6	11.1
Cancer (except skin) (n = 253)	40.9	9.9	47.6	10.6
Depression (n = 942)	45.4	11.6	36.3	11.9
Diabetes (n = 1011)	41.1	11.2	47.8	11.5
Heart disease (n = 691)	38.9	10.0	48.3	10.7
Limited use of arm(s)/leg(s) (n = 605)	39.0	11.5	46.7	12.2
Lung disease (n = 328)	38.3	10.8	45.6	11.5
Osteoarthritis (n = 1013)	38.6	10.1	48.0	10.9
Rheumatoid arthritis (n = 514)	40.0	10.6	47.8	11.3
Vision impairment (n = 628)	44.0	11.6	45.8	11.9

^a ^SD = Standard Deviation

**Table 5:** SF-36v2™ results in ME/CFS patients according to conformity to the Canadian diagnostic criteria

SF-36 scales and summaries	Patients with Canadian diagnostic criteria						Patients without Canadian diagnostic criteria							
	Males (n = 20)		Females (n = 76)		Total (n = 96)		Males (n = 15)		Females (n = 55)		Total (n = 70)		Comparison of total mean scores	
	mean	SD^a^	mean	SD^a^	mean	SD^a^	mean	SD^a^	mean	SD^a^	mean	SD^a^	Mean difference^1^	*P*- value^2^
Physical Component Summary	26.2	7.0	23.5	7.1	24.1	7.2	34.5	8.1	25.8	8.1	30.6	8.7	6.5	0.000
• Physical Functioning	26.9	11.6	24.1	8.2	24.7	9.0	36.2	13.3	30.4	10.7	31.8	11.5	7.1	0.000
• Role-Physical	21.1	5.1	22.9	5.2	22.5	5.2	30.2	10.7	29.1	9.9	29.3	10.0	6.8	0.000
• Bodily Pain	29.4	8.6	30.0	8.3	29.9	8.4	38.1	10.7	33.2	10.2	34.3	10.3	4.4	0.002
• General Health	23.9	6.5	26.6	6.6	26.0	6.6	32.7	8.7	30.6	8.5	31.0	8.5	5.0	0.000
														
Mental Component Summary	25.6	11.2	34.3	11.4	32.5	11.9	35.7	10.4	35.1	11.0	36.0	10.4	3.5	0.023
• Vitality	24.3	6.1	27.3	6.3	26.6	6.3	28.8	7.2	31.5	7.4	30.9	7.4	4.3	0.000
• Social Functioning	20.5	7.1	23.7	8.0	23.3	7.9	32.8	12.3	28.4	10.8	29.4	11.2	6.1	0.000
• Role-Emotional	24.1	17.3	31.7	14.6	29.6	15.3	33.1	15.0	33.0	14.5	33.1	14.5	3.5	0.076
• Mental Health	28.5	12.4	36.2	12.0	34.6	12.4	40.6	12.8	38.9	11.3	39.3	11.6	4.7	0.007

^a^SD = Standard Deviation

^1 ^Patients without Canadian criteria - Patients with Canadian criteria

^2 ^t-test comparing totals of SF-36v2TM in patients, who conform or not with Canadian diagnostic criteria

### Carers

**Figure 2:**
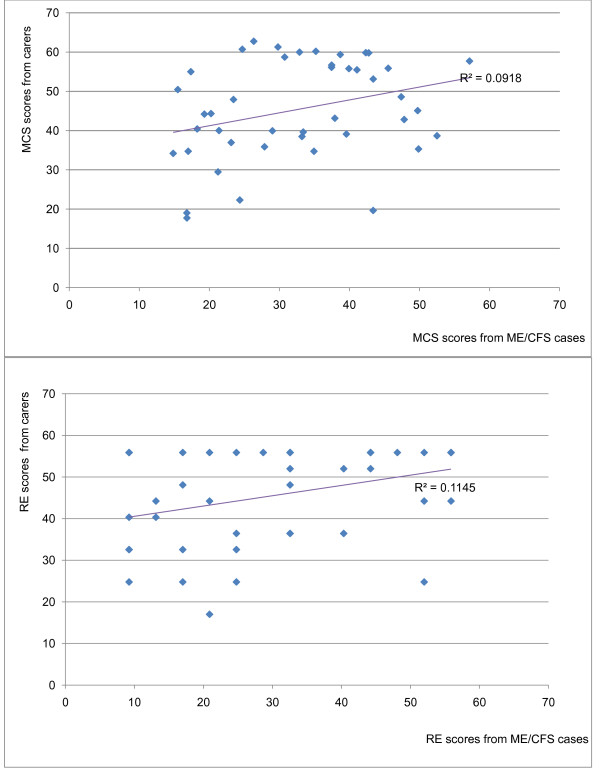
**"Mental Health Summary" and "Role-Emotional" scores from ME/CFS cases and their carers**. MHS - Mental Health Summary; RE - Role-Emotional.

Of the 118 carers named by the patients, 51 responded (43.2% response rate). We were able to match 43 carers to ME/CFS cases. The median age of carers was 53.2 years (IR = 45.1 - 61.7); 52.7 years (IR = 46.4 - 58.8) in men, and 55.1 (IR = 45.2 - 64.3) years in women (*P *= 0.34). Table 6 summarises scores for carers, their paired cases and the 45-54 age population norms. The mean physical component score was within the age norm for the general population, but mental component scores were lower than age norms. The scores for scales within the physical domain were within the age population norms, except for 'General Health', which was over 3 points lower. On the other hand the scores for the scales within the mental domain were consistently lower in the carers, in comparison with age norms. Table 7 shows the results of the regression analysis comparing scores between cases and their carers, showing significant associations in respect of the Mental Component Summary score and 'Role-Emotional' (Figure 2). Table 8 shows that the scores within the mental health domain were consistently lower in women carers than in male carers; this was statistically significant for Vitality (*P *= 0.01) and Mental Health (*P *= 0.03).

**Table 6:** SF-36v2™ results of carers, ME/CFS cases and standard general population norms

SF-36 scales and summaries	Carer's scores		Paired case's scores		General population 45-54 years-old's scores	
	Mean	SD^a^	Mean	SD^a^	Mean	SD^a^
Physical Component Summary	49.1	9.5	25.7	9.1	49.7	9.1
• Physical Functioning	48.9	10.3	26.3	11.1	50.1	8.7
• Role-Physical	47.8	10.0	23.2	6.4	50.4	8.9
• Bodily Pain	50.1	10.8	32.3	11.8	49.3	9.1
• General Health	45.6	12.1	27.3	8.5	49.8*	9.4
Mental Component Summary	45.4	12.6	32.6	11.6	50.6*	9.0
• Vitality	46.1	12.0	26.4	6.0	50.6*	9.2
• Social Functioning	46.3	12.6	22.6	9.3	50.1*	9.2
• Role-Emotional	45.7	11.7	30.6	16.0	50.8*	8.8
• Mental Health	47.1	12.5	35.8	12.9	50.2*	7.9

^a^SD = Standard Deviation

*Minimally important differences in scores observed between carers and general population scores

**Table 7:** Association between SF-36v2™ mean scores in ME/CFS cases and their respective carers

SF-36 scales and summaries	Linear regression analysis results	
	
	*P *value	Adjusted R-squared
Physical Component Summary	0.44	0.009
• Physical Functioning	0.89	0.024
• Role-Physical	0.88	0.024
• Bodily Pain	0.74	0.022
• General Health	0.84	0.023
• Mental Component Summary	0.03	0.080
• Vitality	0.23	0.011
• Social Functioning	0.72	0.021
• Role-Emotional	0.03	0.093
• Mental Health	0.10	0.041

^a^SD = Standard Deviation

**Table 8:** SF-36v2™ norm-based results in carers of ME/CFS patients and age-gender norms (n = 43)

	Males (n = 25)				Females (n = 18)				
SF-36 scores	Carers mean	SD^a^	Age norm mean mean	SD^a^	Carers mean	SD^a^	Age norm mean mean	SD^a^	*P*-value^1^
Physical Component Summary	50.3	7.7	50.9	9.5	47.4	11.6	48.5	8.7	0.161
• Physical Functioning	50.7	7.9	51.5	8.9	46.3	12.7	48.7	8.4	0.081
• Role-Physical	49.7	7.6	51.3	9.6	45.3	12.4	49.6	8.4	0.079
• Bodily Pain	50.4	10.6	50.5	9.9	49.5	11.3	48.2	8.5	0.607
• General Health	47.5	12.5	50.4	10.2	43.0	11.3	49.3	8.8	0.113
Mental Component Summary	47.9	13.4	50.9	10.3	41.8	10.9	50.2	8.1	0.060
• Vitality	49.7	11.8	51.7	10.4	41.0	11.0	49.4	8.3	0.008
• Social Functioning	47.9	12.6	50.6	10.4	44.1	12.5	49.7	8.4	0.168
• Role-Emotional	46.7	11.8	51.3	9.6	44.2	11.7	50.3	8.2	0.249
• Mental Health	50.2	12.9	50.8	10.5	43.0	11.2	49.6	8.3	0.026

^a^SD = Standard Deviation; ^1 ^t-test for comparing carers scores by gender;

## Discussion

ME/CFS is a disabling condition with a high impact on individuals and society, which causes a substantial economic burden [28]. We measured the functional status and well being of a well characterised sample of individuals with ME/CFS and their careers, using SF-36, a widely used and well-validated instrument, which provides generic (i.e. universally-valued, and not specific to age, disease or condition or treatment) measures of disease impact on physical, physiological, social functioning and roles [11] Generic instruments best capture the 'total burden of disease' by expressing the impact of the disease on functioning and well being. Therefore, unlike disease-specific measures, they can be adequately used for comparisons between people with ME/CFS and healthy individuals and those with a range of other diseases [29]. While SF-36 version 2 represents an improvement on its predecessor, it is still meaningful to compare results which use different versions of this instrument [11].

The use of norm-based scores allowed comparisons between scales and component summaries, and helped identify which aspects of quality of life are most affected. The physical and mental health summary measures provide convenient ways to quantify physical and mental health status. Individual-based scores below 40 and group mean scores below 47 may indicate impairment of function. For example, low scores in the physical component scale may relate to limitations in self-care and reduced well-being. Low scores in the mental component scale may relate to frequent psychological distress, and emotionally-driven social and role disability [11].

The scores for the Physical and Mental Health Component summaries and the scales within each of these domains were considerably and consistently lower in people with ME/CFS, when contrasted with individuals with a range of other chronic diseases. By using normalized scores, we were able to show that the scores within the physical domain were even lower than those in the mental domain, although all scales showed very low values. This demonstrates that ME/CFS is not only physically disabling, but also has a significant impact on mental health.

Physical Health Component summaries at the levels found in our study (mean score 26.8) have been shown to relate to an inability to climb one flight of stairs in over 90% of those with similar scores, with three-quarters of those with this scoring level having difficulties at work, with over half needing to reduce the amount of time spent at work, and two thirds rating their health as 'fair' or 'poor' [11]. This score corresponds to around the 5^th ^percentile of the US general population, and is lower than the 25th percentiles of 10 other chronic diseases we used for comparison purposes. The lowest observed mean score was for 'Role-Physical', indicating limitations in functionality for these patients. The values found for this scale are comparable to the 25th percentiles of those with limited use of arm(s) and leg(s), and are lower than the 25^th ^percentiles of those with 9 other conditions.

The mean Mental Health Component summary of 34.1 is comparable to the mean score of those with depression (MID lower than 3), and to the 25^th ^percentile of those with lung disease. It is lower than the 25^th ^percentile of all the other comparison conditions, and corresponds to around the 9^th ^percentile of the general population. The highest values in this domain were for the 'Mental Health' scale, but these were still very low, equivalent to around the 10^th ^percentile of the general population.

The 'Role-Physical' scale was one the most affected of all, suggesting this could be a suitable outcome measure in ME/CFS. The vitality scale has been widely used in ME/CFS research, as it is directly related to the perception of low energy levels typical of those with chronic fatigue. This scale has often presented strikingly low results in people with ME/CFS, on a '0 to 100' score, and this might have helped to reinforce its suitability as an outcome measure in this disorder. In our study, this was 15.5, representing the lowest of all scores. Although still low when normalized scores were used, the 'Vitality' score ranked as the 4^th ^highest score among the 8 health domains scales (the highest score was for Mental Health). This indicates that lack of energy in itself may well not be the most disabling feature of ME/CFS. It also illustrates the inappropriateness of the '0 to 100' score for comparing scales. 'Vitality' scores have inherently low values, as illustrated by the finding for the US general population, where the mean for this score is 58.3. In comparison, the mean score for 'Role-Pyia' in the same population is 82.5, with scores for the remaining scales varying between 75.0 ('Mental Health') and 87.4 ('Role-Emotional').

We have shown that the quality of life of those caring for people with ME/CFS is also affected. In most cases, their scores were lower than those of healthy individuals of the same age group. Interestingly, the Mental Health Component summary and scales within this domain were more sharply reduced, compared with the summary and scales within the Physical Component. In addition, the significant correlations demonstrated between the Mental Health Summary and 'Role-Emotional' scores of patients and their carers suggest that those patients who are less able to carry out emotional roles and whose mental health is more affected represent a greater burden to their carers. These findings may demonstrate the intensity of the emotional pressures on those caring for people with ME/CFS.

Previous studies have shown that a considerable impact on the functional status and well-being or the quality of life of people with ME/CFS [12-14,16,17,19,21,24,25,30-32]. These studies varied in relation to the methods used, including the reference population, how cases were ascertained, and how quality of life has been measured. When SF-36 was used, the scales scores were not normalized, which made comparisons difficult. However, we have also presented our results using the standard scoring system (0-100 scores), to enable comparisons with these previous studies. While low scores were consistently found previously [19,22,24,25,33], they were not as low as in our study. Possible explanations, other than differences in populations and methods, include the specificity of the case definitions we used, which might have excluded cases that would have been positive if other, more complacent diagnostic criteria were used. The fact that the scores of cases meeting the Canadian criteria were consistently lower than those not meeting the criteria further suggests that diagnosis specificity is related to disease severity, and that diagnostic criteria such as the Canadian may be more appropriate for research studies investigating risk factors and disease biomarkers.

### Study strengths and limitations

Our study strengths include the large sampling frame, selection of participants from wide geographic areas, well characterised patients, standardised recruitment procedures and the use of a well validated instrument to measure functional status and well being. The response rate for carers was not particularly high, but all scores were similar in patients whose carers completed the SF-36 and those who did not (data not shown), giving some indication that participation was unlikely to have selected a particular sub-group of carers. Our comparisons were made with references based on the US population. Although comparisons would ideally have been made with the population in the same UK regions, large US studies provide reliable and readily available population norms, including for the general population, specific diseases, gender, and age groups. In addition, the SF-36 instrument has been widely available internationally [11], including in the UK [34], where general population scores have been similar and in some cases slightly higher than those in the US. The comparisons of scores with those with other chronic diseases and the healthy population were based on different age groups, i.e. 18-64 years in our study and 18 and over in the SF-36 population survey. As we would expect population scores to be increased by the exclusion of elderly individuals, restriction of the comparison to those under 65 only would, if anything, tend to show a more dramatic contrast between those with ME/CFS and other disease population groups. The large differences between ME/CFS patients and those in other groups reassure us that the differences are genuine, and would be expected to remain if we used controls from the same geographical area.

The results of the study highlight the disabling nature of ME/CFS. However, the lack of biomarkers and the fluctuating nature and lack of specificity of symptoms makes disease characterisation and disability assessment challenging. Our study supports the potential value of SF-36 as an instrument to characterise incapacity in people with ME/CFS, and particularly that of specific scales, such as the 'Role-Physical'. They may represent a reliable outcome measure indicating case severity for use in observational and interventional studies. However, as the scales are based on patient report, they should ideally be used in combination with other instruments providing objective outcome measurements of physical [35] and neuro-cognitive abilities [36]. A good example of an objective measure of disability is that of cardiopulmonary exercise testing with measurement of VO_2 _max, anaerobic threshold and maximal heart rate and respiration. This test has shown abnormal results in people with ME/CFS [35], and could perhaps be used more often in disability assessments, in combination with instruments based on patient report such as the SF-36.

## Conclusions

Quality of life is inversely related to distress, disability and loss of function, and is associated with the ability of individuals to remain active and perform roles in society. A major goal of people with chronic diseases is to achieve effectiveness in life and to preserve function and well-being. However, people with ME/CFS are by and large failing to achieve these goals, and their carers' emotional well being is also being affected.

Disability assessment in PWME remains a challenge, as the disabling nature of the condition is not always immediately apparent. Nevertheless, recognition of the level of disability faced by these individuals is essential for planning support services that adequately meet their needs. Measures of quality of life outcomes are also essential both for clinical practice and research, particularly in the assessment of interventions. Generic instruments such as SF-36 and individual scales such as 'Role-Physical' may provide meaningful ways to assess the functional ability and wellness of people with ME, especially when combined with objective measures of functional status, thus enhancing the capacity to address the burden of disability experienced by patients and carers.

## List of abbreviations

FMS: Fibromyalgia or fibromyalgia syndrome; FS: Fatigue syndrome; IR: Interquartile range; MCS: Mental Component Summary; ME/CFS: Myalgic encephalomyelitis/chronic fatigue syndrome MID: Minimally Important Difference; MREC: Multi-Centre Research Ethics Committee; NBS: Norm-based score; PCS: Physical Component Summary; PIE: Post-infectious encephalitis; PVAS: Post-viral asthenic syndrome; PWME: People with ME/CFS; SD: Standard deviation; SF-36 v2™: Short form health survey version 2; VO_2 _max: Volume of maximal oxygen consumption

## Competing interests

The authors declare that they have no competing interests.

## Authors' contributions

LN, EL and DP conceived the study and served as principal investigators. LN and EL analysed and interpreted the data and wrote the manuscript. All authors contributed to data collection, interpretation of findings and review of the manuscript; and all authors have read and approved the final manuscript.

## Pre-publication history

The pre-publication history for this paper can be accessed here:

http://www.biomedcentral.com/1471-2458/11/402/prepub

## References

[B1] Baker R Shaw EJ Diagnosis and management of chronic fatigue syndrome or myalgic encephalomyelitis (or encephalopathy): summary of NICE guidance Bmj 2007 335 446 448 10.1136/bmj.39302.509005.AE 17762037 PMC1962830

[B2] Carruthers B Jain AK De Meirleir KL Peterson DL Klimas NG Lerner AM Bested AC Flor-Henry P Joshi P Powles AP Sherkey JA van de Sande MI Myalgic encephalomyelitis/chronic fatigue syndrome: clinical working case definition, diagnostic and treatment protocols Journal of chronic fatigue syndrome 2003 11 7 115

[B3] Fukuda K Straus SE Hickie I Sharpe MC Dobbins JG Komaroff A The chronic fatigue syndrome: a comprehensive approach to its definition and study. International Chronic Fatigue Syndrome Study Group Ann Intern Med 1994 121 953 959 7978722 10.7326/0003-4819-121-12-199412150-00009

[B4] WHO International Classification of Functioning, Disability and Health (ICF) 2001 Geneva: World Health Organization

[B5] Pinquart M Sorensen S Differences between caregivers and noncaregivers in psychological health and physical health: a meta-analysis Psychol Aging 2003 18 250 267 12825775 10.1037/0882-7974.18.2.250

[B6] Pheby D Lacerda E Nacul L Drachler MD Campion P Howe A Poland F Curran M Featherstone V Fayyaz S Sakellariou D de Carvalho Leite JC A Disease Register for ME/CFS : Report of a Pilot Study BMC Res Notes 2011 4 139 10.1186/1756-0500-4-139 21554673 PMC3118997

[B7] Horton SM Poland F Kale S Drachler Mde L de Carvalho Leite JC McArthur MA Campion PD Pheby D Nacul L Chronic fatigue syndrome/myalgic encephalomyelitis (CFS/ME) in adults: a qualitative study of perspectives from professional practice BMC Fam Pract 2010 11 89 10.1186/1471-2296-11-89 21078171 PMC2994803

[B8] Drachler M de L Leite JC Hooper L Hong CS Pheby D Nacul L Lacerda E Campion P Killett A McArthur M Poland F The expressed needs of people with chronic fatigue syndrome/myalgic encephalomyelitis: a systematic review BMC Public Health 2009 9 458 10.1186/1471-2458-9-458 20003363 PMC2799412

[B9] Lacerda EM Nacul L Pheby D Shepherd C Spencer P Exploring the feasibility of establishing a disease-specific post-mortem tissue bank in the UK: a case study in ME/CFS J Clin Pathol 2010 63 1032 1034 10.1136/jcp.2010.082032 20924033

[B10] Osoba T Pheby D Gray S Nacul L The Development of an Epidemiological Definition for Myalgic Encephalomyelitis/Chronic Fatigue Syndrome Journal of Chronic Fatigue Syndrome 2008 14 61 84

[B11] Ware JE Jr Kosinski M Bjorner JB Turner-Bowker DM Gandek B Maruish ME User's manual for the SF-36v2™ health survey 2007 Lincoln: QualityMetric Incorporated21633495

[B12] Buchwald D Pearlman T Umali J Schmaling K Katon W Functional status in patients with chronic fatigue syndrome, other fatiguing illnesses, and healthy individuals Am J Med 1996 101 364 370 10.1016/S0002-9343(96)00234-3 8873506

[B13] Buskila D Fibromyalgia, chronic fatigue syndrome, and myofascial pain syndrome Curr Opin Rheumatol 2001 13 117 127 10.1097/00002281-200103000-00005 11224736

[B14] Hardt J Buchwald D Wilks D Sharpe M Nix WA Egle UT Health-related quality of life in patients with chronic fatigue syndrome: an international study J Psychosom Res 2001 51 431 434 10.1016/S0022-3999(01)00220-3 11516765

[B15] Herrell R Goldberg J Hartman S Belcourt M Schmaling K Buchwald D Chronic fatigue and chronic fatigue syndrome: a co-twin control study of functional status Qual Life Res 2002 11 463 471 10.1023/A:1015635113159 12113393

[B16] Kennedy G Abbot NC Spence V Underwood C Belch JJ The specificity of the CDC-1994 criteria for chronic fatigue syndrome: comparison of health status in three groups of patients who fulfill the criteria Ann Epidemiol 2004 14 95 100 10.1016/j.annepidem.2003.10.004 15018881

[B17] Komaroff AL Fagioli LR Doolittle TH Gandek B Gleit MA Guerriero RT Kornish RJ Ware NC Ware JE Bates DW Health status in patients with chronic fatigue syndrome and in general population and disease comparison groups Am J Med 1996 101 281 290 10.1016/S0002-9343(96)00174-X 8873490

[B18] Lin JM Brimmer DJ Maloney EM Nyarko E Belue R Reeves WC Further validation of the Multidimensional Fatigue Inventory in a US adult population sample Popul Health Metr 2009 7 18 10.1186/1478-7954-7-18 20003524 PMC2801470

[B19] Myers C Wilks D Comparison of Euroqol EQ-5D and SF-36 in patients with chronic fatigue syndrome Qual Life Res 1999 8 9 16 10457734 10.1023/a:1026459027453

[B20] Nijs J Thielemans A Kinesiophobia and symptomatology in chronic fatigue syndrome: a psychometric study of two questionnaires Psychol Psychother 2008 81 273 283 10.1348/147608308X306888 18644213

[B21] Rakib A White PD Pinching AJ Hedge B Newbery N Fakhoury WK Priebe S Subjective quality of life in patients with chronic fatigue syndrome Qual Life Res 2005 14 11 19 10.1007/s11136-004-1693-y 15789937

[B22] Reeves WC Wagner D Nisenbaum R Jones JF Gurbaxani B Solomon L Papanicolaou DA Unger ER Vernon SD Heim C Chronic fatigue syndrome - a clinically empirical approach to its definition and study BMC Med 2005 3 19 10.1186/1741-7015-3-19 16356178 PMC1334212

[B23] Wagner D Nisenbaum R Heim C Jones JF Unger ER Reeves WC Psychometric properties of the CDC Symptom Inventory for assessment of chronic fatigue syndrome Popul Health Metr 2005 3 8 10.1186/1478-7954-3-8 16042777 PMC1183246

[B24] Posner N Madl R Siskind V Free University of Brussels Pattern of functional impairment in CFS Second world congress on chronic fatigue syndrome and related disorders; Brussels 1999

[B25] Smits M van Rooy R Nagtegaal J Influence of melatonin on quality of life in patients with chronic fatigue syndrome and late melatonin onset Journal of chronic fatigue syndrome 2002 10 25 36 10.1300/J092v10n03_03

[B26] Saris-Baglama R Dewey C Chisholm G Plumb E Kosinski M Bjorner JB QualityMetric Health Outcomes™ scoring software 2.0 user's guide 2007 Lincoln: QualityMetric Incorporated20852675

[B27] Kirkwood BR Sterne JAC Medical statistics 2003 2 Oxford: Blackweel Science

[B28] Jason LA Benton MC Valentine L Johnson A Torres-Harding S The economic impact of ME/CFS: individual and societal costs Dyn Med 2008 7 6 10.1186/1476-5918-7-6 18397528 PMC2324078

[B29] Ware JE Jr Kosinski M Bjorner JB Turner-Bowker DM Gandek B Maruish ME User's manual for the SF-36v2TM health survey 2007 Lincoln: QualityMetric Incorporated21633495

[B30] Anderson JS Ferrans CE The quality of life of persons with chronic fatigue syndrome J Nerv Ment Dis 1997 185 359 367 10.1097/00005053-199706000-00001 9205421

[B31] Schweitzer R Kelly B Foran A Terry D Whiting J Quality of life in chronic fatigue syndrome Soc Sci Med 1995 41 1367 1372 10.1016/0277-9536(95)00124-P 8560304

[B32] Solomon L Nisenbaum R Reyes M Papanicolaou DA Reeves WC Functional status of persons with chronic fatigue syndrome in the Wichita, Kansas, population Health Qual Life Outcomes 2003 1 48 10.1186/1477-7525-1-48 14577835 PMC239865

[B33] Jason L Brown M Evans M Anderson V Lerch A Brown A Hunnell J Porter N Measuring substantial reductions in functioning in patients with chronic fatigue syndrome Disabil Rehabil 2011 10.3109/09638288.2010.503256 PMC3170036 20617920

[B34] Jenkinson C Stewart-Brown S Petersen S Paice C Assessment of the SF-36 version 2 in the United Kingdom J Epidemiol Community Health 1999 53 46 50 10.1136/jech.53.1.46 10326053 PMC1756775

[B35] Make B Jones JF Impairment of patients with chronic fatigue syndrome Journal of chronic fatigue syndrome 1997 3 43 55

[B36] Dimitrov M Grafman J Neuropsychological assessment of chronic fatigue syndrome Journal of chronic fatigue syndrome 1997 3 31 42 10.1300/J092v03n04_05

